# Aspirin Prophylaxis for the Prevention of Thrombosis: Expectations and Limitations

**DOI:** 10.1155/2012/104707

**Published:** 2012-02-07

**Authors:** Gundu H. R. Rao, Jawad Fareed

**Affiliations:** ^1^Lillehei Heart Institute, University of Minnesota, 420 Delaware Street SE, Minneapolis, MN 55455, USA; ^2^Departments of Pathology and Pharmacology, Loyola University Medical Center, 2160 South First Avenue, Maywood, IL 60153, USA

## Abstract

Platelets play a very important role in the pathogenesis of acute vascular events leading to thrombosis of the coronary and cerebral arteries. Blockage of these arteries leading to regional ischemia of heart and brain tissues precipitate heart attacks and stroke. Acetyl salicylic acid (Aspirin) has been the drug of choice for over half a century for the primary and secondary prophylaxis of thrombotic events. In spite of its extensive use as an antiplatelet drug for the prevention of vascular thrombosis, there is considerable concern about the degree of protection it offers, to patients under aspirin therapy. In this paper, we explain the phenomenon of aspirin resistance, discuss the limitations of aspirin therapy, and suggest methods to monitor “at-risk” individuals. Ability to monitor and determine at risk patients will provide opportunities for the clinicians to customize antiplatelet therapies.

## 1. Introduction

Role of platelets in the pathogenesis of thrombosis and stroke is well documented [1]. There is a great need for developing specific and effective antiplatelet drugs for modulating platelet function. A thorough understanding of the signaling mechanisms involved in the regulation of platelet function will facilitate the development of better antiplatelet drugs. Agonists interact with the platelet at specific receptor sites on the plasma membrane and initiate a series of signaling events capable of modulating shape change, adhesion, aggregation, secretion of granule contents, and expression of activation markers on the membrane [2, 3]. Platelet aggregates are formed when the GP11b/111a receptors get activated and bind fibrinogen and recruit other platelets to form clumps of activated cells. This phenomenon of platelet activation plays a significant role in the formation of effective haemostatic plug as well as the growth of thrombus. Weak agonists such as epinephrine, ADP require the production of proaggregatory prostaglandin (PG) endoperoxides and thromboxane to cause platelet aggregation and secretion. Aspirin is a specific inhibitor of cyclooxygenase (COX-1) and prevents the formation of pro-aggregatory PG endoperoxides.

 Data from large number of clinical studies have demonstrated that at any given risk for the development of acute vascular events, irrespective of the disease state, aspirin at low-to-medium concentration is as effective as any other drug in reducing the risks [4]. Although ability of aspirin to reduce fever was discovered two hundred years ago, the mechanism of action of aspirin remained elusive till late 1900 [5]. Nobel laureate Sir John R. Vane and his associates in 1971 proposed the mechanism as to how aspirin works [6, 7]. Within a short period of time extensive work was done by various groups to elucidate the mechanism of action of aspirinlike compounds [8–20]. At the same period, another Nobel Laureate Dr. Bengt Samuelsson discovered that the prostaglandin synthase produces transient bioactive prostanoids like PGG_2_/PGH_2_ and thromboxane A_2_ from the substrate arachidonic acid [9]. These findings revolutionized the research in platelet physiology and pharmacology [2–21].

## 2. Platelet Physiology

Blood platelets interact with a variety of soluble agonists such as epinephrine (EPI), adenosine diphosphate (ADP), thrombin, and thromboxane (TXA_2_) and many cell matrix components, including collagen, laminin, fibronectin, and von Willebrand factor and biomaterials used for construction of invasive medical devices [21–26]. These interactions stimulate specific receptors and glycoprotein-rich domains (integrin and nonintegrin receptors) on the plasma membrane and lead to the activation of intracellular effector enzymes. Agonist–mediated activation of platelets stimulates phospholipase C (PLC) and induces the hydrolysis of Phosphatidyl inositol 4, 5-bisphosphate and formation of second messengers 1, 2-diacyl glycerol and inositol 1, 4, 5 trisphosphate. Diacylglycerol activates protein kinase and inositol trisphosphate facilitates the mobilization of free calcium from the storage sites. The majority of regulatory events appear to require free calcium. Ionized calcium is the primary bioregulator, and a variety of biochemical mechanisms modulate the availability of free calcium [26]. Elevation of cytosolic calcium stimulates phospholipase A_2_ and liberates arachidonic acid (AA) from platelet membrane phospholipids. Free AA is transformed to a novel metabolite thromboxane, a potent platelet agonist. This is the major metabolite of AA metabolism that plays an important role in platelet recruitment, granule mobilization, secretion of granule contents, and expression of activated GP11b/111a (ά_11b_ *β*_3_) receptors [21–28]. Upregulation of activation signaling pathways will increase the risk for clinical complications associated with thrombotic events.

## 3. Arachidonic Acid Metabolism

Arachidonic acid is a 20-carbon polyunsaturated fatty acid (20:4w6), found in platelet membrane phospholipids. Platelet activation stimulates Phospholipase A_2_, which facilitates the release of this fatty acid from membrane phospholipids. AA is converted to prostaglandin (PG) endoperoxides (PGG_2_/PGH_2_) by cyclooxygenase (Prostaglandin G/H synthase; COX1). These metabolites are converted by thromboxane synthetase to thromboxane A_2_, which is the major metabolite of this pathway in platelets [9]. Whereas, in vascular tissues, the endoperoxides generated by COX1 are transformed by prostacyclin synthetase to prostacyclin (PGI_2_). Thromboxane is a potent platelet agonist and a vasoconstrictor. Prostacyclin is an antiplatelet compound and exerts vasodilatory effects on vascular tissues. Thus from a single substrate (AA), two pharmacologically opposing vasoactive prostanoids are generated by platelets and vascular tissues [2, 3]. Aspirin selectively acetylates the hydroxyl groups of a single serine residue (position 529) in the prostaglandin G/H synthase and causes irreversible inhibition of the activity of this enzyme [11, 12]. Inhibition of PG synthase results in the decreased conversion of AA to PG endoperoxides, PGG_2_/PGH_2_. Molecular mechanisms involved in aspirin-mediated inhibition of prostaglandin G/H synthase are well documented [3, 14].

## 4. Studies on the Use of Aspirin as an Inhibitor of Cyclooxygenase Enzymes

Single oral doses of 10–100 mgs of aspirin can significantly inhibit the platelet PG synthase activity [29]. The inhibitory effect of aspirin on circulating platelets in the blood is for a very limited time and probably occurs in the portal circulation. The half-life of aspirin is very short (15–20 minutes), but sufficient to inhibit PG synthase of circulating platelets. Since these cells lack DNA and the ability to resynthesize the enzyme, the dysfunction caused by aspirin cannot be overcome. Therefore, platelets exposed to aspirin loose the ability to make the prostanoids completely. However, one should keep in mind that once the aspirin is hydrolyzed to salicylic acid, ability to inhibit prostaglandin synthase is lost. Hence the platelets produced from the marrow after the aspirin is hydrolyzed will have active prostaglandin synthase. Approximately 10% of fresh platelets are added on to the circulating blood every day. Although aspirin-treated blood platelets do not make prostaglandins, they respond with aggregation to the stimulation by prostaglandin endoperoxides and thromboxane. Fresh platelets formed after the hydrolysis of aspirin can synthesize prostanoids and these newly formed metabolites of AA can cause aggregation of aspirin exposed platelets. In view of the fact that aspirin irreversibly inhibits prostaglandin synthase, it is possible to take advantage of repeated low-dose aspirin to achieve a cumulative effect [29–42]. Even doses as low as 30–50 mg aspirin taken daily will suppress platelet thromboxane synthesis significantly in 5 to 10 days. Vascular tissues on the other hand have the ability to resynthesize prostaglandin G/H synthase [29]. Therefore, these cells can recover the enzyme activity following aspirin exposure. It is, therefore, possible to develop a strategy to promote the biochemical selectivity of aspirin in terms of inhibition of platelet prostaglandin synthase. This is done by modification of the drug delivery, so the amount of drug delivered is just enough to inhibit platelet enzymes in the peripheral circulation and spare the systemic effect on vascular endothelium [30, 31]. Several studies have demonstrated the feasibility of this approach and various control release or timed release formulations have been developed for this novel therapy [30–32].

As mentioned earlier, aspirin is metabolized rapidly and the major metabolite, salicylic acid, is a poor inhibitor of platelet prostaglandin synthase. Therefore, it is essential to develop appropriate strategies to maximize the beneficial effect of this novel drug. As low-dose as 20 mg taken daily reduces the platelet thromboxane formation by more than 90 percent. However, it is generally believed that higher doses are essential for preventing thromboxane-dependent platelet activation. Studies by Wilson et al. demonstrated that maximal plasma concentration of 12 umol/L could be achieved by a single oral 50 mg dose of enteric-coated aspirin [16]. This dose was found sufficient to cause significant inhibition of platelet function and daily ingestion of low-dose aspirin demonstrated a cumulative effect. In a separate study, McLeod et al. used doses ranging from 50 to 3900 mg of aspirin and monitored platelet function, bleeding time, and concluded that maximum dysfunction was obtained with daily doses of about 100 mg and no further changes were observed in these studies with higher doses [17]. Several workers have demonstrated the efficacy of low-dose oral aspirin in preventing platelet thromboxane production [2–4, 17, 43]. Indeed one of these studies has demonstrated beneficial effect of a dermal aspirin preparation on selective inhibition of platelet prostaglandin synthase, sparing the prostacyclin biosynthesis [31]. It is very well established that 100 mg of aspirin per day is sufficient to significantly reduce the platelet thromboxane production [2–4, 19, 20, 33–35]. Furthermore, studies by McLeod et al. have shown that dosages higher than 100 mg per day do not produce any greater inhibition of platelet function or enhance bleeding times [17]. Therefore, it is reasonable to conclude that 80–160 mgs aspirin per day should be the choice for an ideal preventive protocol [33]. However, there is considerable room for improvement to maximize the benefits by better understanding the pharmacology of aspirin and platelet physiology [2–4]. It is possible to customize the aspirin treatment based on the individual patient needs. One can monitor the platelet prostaglandin synthase activity following aspirin ingestion and recommend a dose that is appropriate [28–34]. It is possible to monitor the platelet response to agonists such as ADP or arachidonate and determine the degree of inhibition by aspirinlike compounds [17]. In order to get maximum inhibition of platelet COX1 enzymes, continuous release aspirin formulations can be developed and tested against currently available aspirin formulations. Platelets are produced and released constantly to the circulation. Therefore, a time-release aspirin, which would make available small amounts of aspirin into the circulation, may be effective. For instance, a 100 mg formulation capable of releasing 10 mg acetyl salicylic acid per hour may be better than a preparation which releases all of its active principle in a short span of time. Using the strategy of slowing down the release of active principle, newer formulations could be used effectively to provide needed amounts of the drug into circulating blood at regular intervals. These novel formulations may also provide selectivity of aspirin action by preventing platelet thromboxane production and sparing the endothelial prostacyclin synthesis. McLeod et al. studied the effect of various doses of aspirin (50, 100, 325, and 1000 mg) on platelet and vascular tissues [36]. They did not observe inhibition of urinary 6-keto-PGF1 alpha production at low-doses of 50 and 100 mg. They attributed these findings to the differential and selective inhibition of platelet function and the sparring effect of vascular COX1 enzymes. Sullivan and associates studied the effect of two different doses of aspirin on platelet function and TXA_2_ production [38]. Platelet function in healthy volunteers was inhibited by both the doses (75 and 300 mg). Low-dose failed to inhibit completely TXB_2_ production 24 hours later, whereas 300 mg aspirin did. Even alternate day regimen of these doses prevented platelet function and significantly inhibited the urinary levels of the 11-keto-TXB_2_. In a separate study, in healthy volunteers, formation of thrombin (Fibrinopeptide A; FPA), alpha granule release (beta-thromboglobulin; beta TG), and thromboxane (TXB_2_) were monitored in vivo, in blood emerging from a template bleeding incision [39]. At the site of plug formation significant platelet activation and thrombin generation was observed as indicated by 110-fold, 50-fold, and 30-fold increase in FPA, beta TG, and TXB_2_, within the first minute. A low-dose regimen (0.42 m/kg/day for 7 days) caused greater than 90% inhibition of TXB_2_ formation in both bleeding time and clotted blood in these studies, suggesting critical role of platelet activation at the site of haemostatic plug formation. In a study to evaluate the effect of low-dose aspirin (0.5 and 15 mg/kg/day) on platelet and renal prostanoids, Wilson et al. monitored serum TXB_2_ and urinary 6-keto PGF1 alpha [40]. Serum TXB_2_ level was reduced to 3% of control by low-dose and to 0.1% by the higher dose. Urinary TXB_2_ was reduced only to 68% by low-dose aspirin and to 51% by high dose. Urinary 6-keto-PGF1 alpha was not reduced by either dose. Based on their observation, they concluded that low-dose aspirin could significantly affect platelet PG production without affecting stimulated release of PGI_2_ production.

## 5. Clinical Studies on the Use of Aspirin

The two major clinical trials on aspirin concluded that ingestion of 160 mg per day or 325 mg alternative day provided significant benefit in preventing fatal events associated with CAD [19, 20]. Whereas, a 10-year trial involving nearly 40,000 women aged 45 and older with no evidence of cardiovascular disease found that a regular alternate day low-dose (100 mg) aspirin was effective in reducing the incidence of stroke, but it did not have any effect on the incidence of heart attacks [41]. They concluded that the reasons for any sex-based differences in the efficacy of aspirin for primary prevention are unclear. According to Minnesota Heart Survey, about 6% of healthy women under age 65 and 30% of those over 65 take low-dose aspirin to prevent acute vascular events [42]. Data from this primary prevention study does not apply to women who already have had a heart attack or heart surgery or diagnosed with coronary artery disease. For such women, as found in men, regular daily low-dose (80–160 mg) of aspirin clearly reduces the risk of developing acute coronary events.

Several earlier studies evaluated the effect of low-dose aspirin on normal healthy volunteers as well as patients with various vascular diseases [38–41]. However, earlier studies did not report prevalence of any aspirin resistance. Zucker et al. evaluated the effect of low-dose aspirin (0.45 mg/kg/day) and a high dose (900 mg/day) in type 11 hyperlipoproteinemic subjects [43]. They found that low-dose aspirin effectively inhibited platelet function in these patients. Increased platelet thromboxane production has been described in several disorders including type 2 diabetes and type 11a hypercholesterolemia. This increased production of TXB_2_ in hypercholesterolemic patients is attributed to abnormal cholesterol levels in these patients. It has been shown that even a low-dose of aspirin (50 mg/7 days) significantly reduces 11-dehydro-TXB_2_, in these patients [44]. The effect of low-dose aspirin has been evaluated in patients with diabetes, coronary heart disease, myocardial infarction (MI), cerebrovascular disease, peripheral artery disease, and a variety of surgical procedures [38–41]. DiMinno et al. studied the effect of single doses of 100 and 1000 mg aspirin for 1 month in normal volunteers and patients with diabetic angiopathy [45]. They found a dose schedule of aspirin, which may suffice in normal volunteers was not effective in patients with diabetic angiopathy. Contrary to this observation, Terres et al. found a low-dose of aspirin (100 mg) caused significant inhibition of platelet function in both healthy subjects and patients with coronary heart disease [46]. Similarly, a low-dose (0.45 mg/kg/day) was found adequate for selective inhibition of TXA_2_-related platelet function, in patients recovering from MI [47]. Looks like the results on the effect of low-dose aspirin vary considerably, depending upon the type and stage of disease, dose of aspirin, and severity of procedure. In a study evaluating the effect of low-dose aspirin (100 mg) on hematological activity of left ventricular (LV) thrombus in anterior wall acute MI (AMI), Kupper et al. found that low dose had no effect on the incidence of hematologic activity and embolic potential of LV thrombosis in anterior wall AMI [48]. On the other hand, a low-dose aspirin (40 mg/day) taken daily was found to be as effective as higher doses in preventing platelet functional responses in patients who had recent cerebral ischemia [49]. Uchiyama et al. evaluated the effect of low-dose aspirin, ticlopidine, and a combination of both these drugs in patients with cerebral ischemia [50]. Aspirin alone markedly inhibited platelet aggregation induced by AA, partially inhibited aggregation induced by ADP and did not inhibit aggregation by platelet activating factor. Combination of these drugs inhibited aggregation by all agonists. Rao et al. demonstrated, in healthy volunteers, that low doses of aspirin (40–80 mg) had no inhibitory effect on the response of platelets to ADP, epinephrine, and thrombin, but effectively inhibited the platelet response to threshold concentrations of AA [2, 3]. Epinephrine at concentrations too low to cause aggregation restored the sensitivity of aspirin-treated platelets to AA [51–54]. This phenomenon, in which weak agonists restore the sensitivity of drug-induced refractory platelets to the action of other agonists, was described from our laboratory as “mechanism of membrane modulation” [51–57].

## 6. Aspirin Resistance

Studies from our laboratory for the first time demonstrated that one could induce drug-mediated resistance in platelets to the action of aspirin [58]. In this study, the subjects were given a short acting inhibitor of COX1, Ibuprofen. This was followed by administration of a full strength (325 mg) aspirin. Ibuprofen-mediated inhibition of COX1 enzyme lasts for a short time, whereas aspirin-induced inhibition is irreversible. Ibuprofen-treated platelets recovered their sensitivity to the action of AA by 24 hrs. Whereas aspirin-treated platelets failed to respond to the action of AA even after 24 hrs. In those subjects who had ingested aspirin after taking Ibuprofen first, aspirin failed to inhibit irreversibly the COX1, suggesting that Ibuprofen molecules effectively prevented the acetylation of COX1enzyme by aspirin. One of the earliest works describing “nonresponders” and “responders” evaluated the effect of low-dose aspirin and a thromboxane synthetase inhibitor dazoxiben (UK3724B) in healthy subjects [59]. These studies demonstrated that low-dose aspirin and ingestion of two dazoxiben tablets prevented the release of granules from platelets in response to AA in some individuals (responders) and not in others (nonresponders). These subtle differences in response of platelets to various drugs as well as differences in response to various agonists may be critical when considering the outcome of acute vascular events. For instance, collagen seems to exert its effect by multiple mechanisms. In a study, using aspirin, monoclonal antibodies to 11b-111a receptor and fibrinogen, it was demonstrated that there exist at least three mechanisms by which collagen activates platelets: (1) GP11b-111a-associated activation, (2) prostaglandin-dependent pathway, and (3) alternate pathway responsible for 20–30% platelet aggregation [60]. 

Several recent studies have demonstrated drug resistance in patients with a variety of vascular diseases [61–95]. This subject currently is a very hot topic and has made national headlines. Andrew Pollack published an article in July of 2004 in New York Times, on this subject titled, “For Some, Aspirin May Not Help Hearts” [62]. According to this article, 5–40% of aspirin users are “nonresponders” or “resistant” to the drug. In the same article, he cites the opinion of Dr. Daniel I. Simon, the associate director of interventional cardiology at Brigham and Women's Hospital, Boston, which reads as follows: “They are taking it for stroke and heart attack prevention and it's not going to work.” He also reports the opinion of Dr. Michael J. Domanski; head of clinical trials unit at the NIH; in his opinion, the nonresponders may represent a huge number of patients. According to Dr. Deepak L. Bhatt, director of interventional cardiology Cleveland Clinic, aspirin resistance is associated with worst outcome. Professor Eric Topol, Chairman, Cardiovascular Medicine Cleveland Clinic, USA states, “Aspirin resistance carries high risk, with over 20 million Americans taking aspirin to prevent heart attacks or strokes, it is important that further work to be done to confirm our findings and develop a rapid detection method. He also assures that for individuals with aspirin resistance, there are excellent alternatives.”

These observances from health care providers and researchers raise number of issues. Do we know enough about aspirin resistance? What is the prevalence of aspirin resistance in healthy population? What causes this resistance to develop in patient populations? Are there specific, rapid, cost-effective tests available? What alternative long-term treatments are available, if patients are resistant to common antiplatelet drugs such as aspirin and Clopidogrel? Should the doses of these drugs used for therapy be increased? Should we use dual antiplatelet therapy or use more than two antiplatelet drugs? Should we drop the use of these drugs in nonresponders? We need to find answers to these and other emerging questions soon. In the next few paragraphs a brief overview of what is known about the prevalence of aspirin resistance, clinical findings, and methodologies available, will be provided.

The first and foremost need at this time is to standardize a definition of aspirin resistance. The mechanism of action of aspirin is very well documented [5–13]. The drug acetylates the platelet COX1 enzyme and irreversibly inhibits its ability to convert AA to PG endoperoxides [11, 12]. In the absence of COX1 enzyme activity, platelets do not respond to AA stimulation with aggregation. Weak agonists such as ADP, Epinephrine depend on the formation of PG endoperoxides to initiate secondary wave of aggregation and promote release of platelet granule contents [2, 3].Therefore, weak agonists fail to induce platelet aggregation and release granules from aspirin-treated platelets. Failure of AA, ADP, and Epinephrine to cause aggregation of platelets more or less establishes drug-induced platelet dysfunction. If platelets obtained from individuals who have ingested a full strength aspirin, respond with aggregation to the action AA, ADP and EPI, and release their granule contents, then one can safely conclude that these platelets are resistant to aspirin action. Further proof for aspirin resistance of platelets can be provided by studying AA metabolism by such platelets, monitoring serum TXB_2_ levels, or urinary levels of TXB_2_ or its metabolite, 11-dehydro-TXB_2_. Methods are available to monitor all these parameters. According to Cattaneo, “aspirin resistant” should be considered as description for those individuals whom aspirin fails to inhibit thromboxane A_2_ production, irrespective of the results of unspecific tests of platelet function [93].

## 7. Prevalence of Aspirin Resistance

Aspirin resistance has been poorly defined, variety of nonspecific methods have been employed to monitor the “aspirin resistance” and conflicting reports have been published on the rates of prevalence and outcome of continuing this therapeutic modality [62–79]. Aspirin resistance has been reported in patients with cardiovascular, cerebrovascular and peripheral vascular disease [75–93]. Because of the differences in methodologies used to monitor this phenomenon and lack of a specific assay to determine the true aspirin resistance, there is considerable confusion and the true significance of this observation remains obscure [62–64]. It also raises the question, as to how we missed this phenomenon of drug resistance all these years? Large numbers of clinical trials have demonstrated the beneficial effects of aspirin therapy irrespective of the disease state [33]. Is it possible that these earlier trials missed aspirin nonresponders? On the other hand, it is quite possible that only responders to the action of aspirin got the benefit of this therapy.

Studies in our laboratory over three decades have failed to show any aspirin resistance in normal healthy subjects. The only subject whose platelets failed to aggregate in response to AA stimulation was found to be deficient in platelet COX1enzyme activity [53]. Platelets obtained from this subject responded with aggregation when stirred with epinephrine and arachidonate, suggesting that PG endoperoxides and TAX_2_ are not essential to cause irreversible aggregation of platelets. There is no much data on the prevalence of aspirin resistance in general healthy subjects. In patients with various vascular diseases, the rate of nonresponders reported varies between less than 2% to over 60%. Since the methods used to monitor aspirin resistance in these reports are not specific, the prevalence rate published is debatable [62–79].

Hurlen et al. used the method of Wu and Hoak to determine the platelet aggregation ratio as a marker for assessing platelet function and evaluated the effect of aspirin (160 mg/day) in 143 patients who had survived myocardial infarction [67, 68]. Based on their definition of nonresponders to the action of aspirin, they could only identify two subjects as primary nonresponders. Gum et al. from Cleveland Clinic studied 326 stable cardiovascular subjects on aspirin (325 mg/day) and tested aspirin sensitivity by platelet response to aggregating agents such as ADP and AA. They found 5.5% as nonresponders to aspirin and 24% as semiresponders [69]. Gum and associates used the PFA-100, a method that measures platelet function, to determine aspirin resistance in their patient population [70]. Based on the results of their studies with this methodology, they found 9.5% to be nonresponders to aspirin action.

Some studies have reported as high as 30–40% nonresponders of stroke or vascular disease patients and predicted >80% increased risk for a repeat event during a 2-year follow-up period [70–74]. Eikelboom et al. analyzed base line urinary levels of TXB_2_ metabolites 11-dehydrothromboxane B2 in 5529 patients enrolled in the Heart Outcomes Prevention Evaluation (HOPE) study [89, 90]. Of these subjects 488 were on aspirin regimen. On the basis of their findings they concluded that in aspirin-treated patients, increased levels of urinary metabolite of TXB_2_ predict future risk of myocardial infarction or cardiovascular death. The patients with the highest levels of urinary TXB_2_ metabolite had 3–5-fold higher risk of cardiovascular death compared to those in the lowest quartile. Another study reporting clinical outcomes of aspirin resistance is from Austria [71, 73, 76]. In this study patients undergoing arterial angioplasty were on 100 mg aspirin per day. Platelet function was assessed by whole blood aggregometry. This study demonstrated that reocculsion at the sites of angioplasty occurred only in men for whom platelet dysfunction was evident by aggregometry [78]. Zimmerman et al. identified aspirin nonresponders as those who had >90% inhibition of TXB_2_ formation in presence of 100 umol/L aspirin and 1 mmol/L arachidonate [78]. In patients who had undergone coronary bypass surgery (CABG), AA and Collagen stimulated formation of TXB_2_ was same before and after CABG, indicating that oral aspirin did not significantly inhibit platelet COX1. However, the in vitro studies with 100 umol/L aspirin on blood obtained from these subjects showed decreased TXB_2_ ( >10%) in most samples studied. They concluded that platelet COX1 inhibition by aspirin is compromised for several days after CABG, probably due to an impaired interaction between aspirin and platelet COX1. This observation indicates how complex the issues are when evaluating the effect of antiplatelet drugs during and after interventional procedures. Sane et al. evaluated the effect of aspirin (325 mg/day/month) in patients suffering from congestive heart failure [79]. These researchers used whole blood aggregometry (Chronolog Corp, PA, USA), Platelet receptor expression by flow cytometry and PFA-100. Patients were considered nonresponders when 4 of the 5 parameters assayed were observed. Using this complex rating, persistent platelet activation was observed in 50 of the 88 patients (56.8%). These observations remind us of the inadequacy of the existing methods to detect what truly represents “aspirin resistance.”

## 8. Aspirin Therapy: Expectations and Limitations

Data from over five hundred clinical studies have demonstrated that at any given risk for the development of acute vascular events, irrespective of the disease state, aspirin at low-to-medium concentration is as effective as any other antiplatelet drug in reducing the risks [4]. In view of these observations, patients as well as physicians who are treating them, expect maximum protection from such an antiplatelet therapy. However, recent studies have demonstrated that close to 30% of the patients on aspirin prophylaxis may be at risk for developing acute vascular events. These revelations have caused considerable confusion in the minds of medical community as well as patients, who are taking antiplatelet drugs. It is important to realize first of all that aspirin is short lived in the circulation and as such only the platelets (COX enzymes), which get exposed to aspirin in the blood during its short period of activity, are permanently inhibited. In addition, bone marrow produces platelets continuously and delivers them into circulating blood; therefore, those platelets that are released after aspirin is hydrolyzed will not have any inhibitory effect of aspirin on their COX-1 enzymes. Studies from our laboratory also have demonstrated that platelets lacking COX-1 enzymes as well as those in which the activity has been inhibited by aspirin respond in a normal fashion and aggregate to the response of arachidonate in the presence of epinephrine, although they do not produce any proaggregatory PG metabolites [35, 51–54, 59]. Studies from our laboratory as well as that of others have demonstrated that patients on aspirin prophylaxis may still produce large amounts of urinary metabolites of pro-aggregatory thromboxane in spite of the fact that COX-1 enzymes of platelets are inhibited in these individuals.

In spite of the high expectations of clinicians as well as patients who are on aspirin prophylaxis, it is reasonable to assume that there is a fair percentage of individuals on antiplatelet drugs who are at risk for developing acute vascular events [90–95]. Therefore, clinicians should assess the risk for these patients by monitoring urinary metabolites of thromboxane, so that if the levels are significantly above normal, then patients have to be provided additional or alternate antiplatelet therapy [95]. Some of the suggested therapies include using instead of single daily dose of aspirin, multiple doses (Eg: 80 mg twice or thrice a day instead of single 80 or 160 mg). Those found with excess of urinary thromboxane levels may be put on thromboxane antagonists. Alternate therapies such use of Triflusal (fluoride derivative of aspirin) or omega three acid capsules can also be tested [96]. If tests to determine urinary metabolites of prostaglandins are not available, then a simple platelet function test with arachidonate as agonists would serve the purpose. Newer instruments are being developed which measure coagulation profile on nonanticoagulated blood PlaCor Platelet reaction testing (PlaCor Inc, Minneapolis, MN) or Aggredyne Platelet function Monitor (Aggredyne, Houston, TX) [97]. Major take-home message, however, when considering the benefits of antiplatelet therapy is that if the platelet functions are not inhibited by the specific antiplatelet drug they are taking, then the patients are “at risk” for acute vascular events. 
